# A Mini Review on Bismuth-Based Z-Scheme Photocatalysts

**DOI:** 10.3390/ma13225057

**Published:** 2020-11-10

**Authors:** Ruizhen Li, Hanyang Chen, Jianrong Xiong, Xiaoying Xu, Jiajia Cheng, Xingyong Liu, Guo Liu

**Affiliations:** 1 School of Chemistry and Environmental Engineering, Sichuan University of Science and Engineering, Huixing Rd, Ziliujing District, Zigong 64300, China; liruizhen007@suse.edu.cn (R.L.); 31808520425@stu.suse.edu.cn (H.C.); 17181020238@stu.suse.edu.cn (J.X.); 17181020239@stu.suse.edu.cn (X.X.); 18181030128@stu.suse.edu.cn (J.C.); 2 State Environmental Protection Key Laboratory of Synergetic Control and Joint Remediation for Soil & Water Pollution, Chengdu University of Technology, No. 1 Dongsan Road, Er’xian Bridge, Chengdu 610059, China; 3 School of Chemical Engineering, Sichuan University of Science and Engineering, Huixing Rd, Ziliujing District, Zigong 64300, China; liuxy@suse.edu.cn; 4 State Key Laboratory of Geohazard Prevention and Geoenvironment Protection, Chengdu University of Technology, No. 1 Dongsan Road, Er’xian Bridge, Chengdu 610059, China

**Keywords:** Z-scheme photocatalysts, bismuth-based semiconductors, environmental remediation, energy production, photocatalytic applications

## Abstract

Recently, the bismuth-based (Bi-based) Z-scheme photocatalysts have been paid great attention due to their good solar energy utilization capacity, the high separation rate of their photogenerated hole-electron pairs, and strong redox ability. They are considerably more promising materials than single semiconductors for alleviating the energy crisis and environmental deterioration by efficiently utilizing sunlight to motivate various photocatalytic reactions for energy production and pollutant removal. In this review, the traits and recent research progress of Bi-based semiconductors and recent achievements in the synthesis methods of Bi-based direct Z-scheme heterojunction photocatalysts are explored. The recent photocatalytic applications development of Bi-based Z-scheme heterojunction photocatalysts in environmental pollutants removal and detection, water splitting, CO_2_ reduction, and air (NO_x_) purification are also described concisely. The challenges and future perspective in the studies of Bi-based Z-scheme heterojunction photocatalysts are discussed and summarized in the conclusion of this mini review.

## 1. Introduction

The rapid growth of industry and the population has resulted in the over-consumption, unreasonable exploitation and utilization of fossil fuel resources. The global energy crisis and environmental deterioration have become two primary challenges of the 21st century in human society [1,2]. Therefore, it is necessary to develop renewable energy sources that can replace fossil fuels. As a powerful and inexhaustible renewable energy source, solar energy is considered as one of the best methods to mitigate these problems. It can be exploited and utilized for energy production (for example H_2_ [3,4,5,6], hydrocarbon fuel [7,8] and electric energy [9,10] production) and removal of pollutants (such as CO_2_ [11,12,13,14,15], organic contaminants in water [16,17,18,19,20,21] or air [22,23,24,25,26], emerging micropollutants [27,28]) by using photocatalytic, photovoltaic and other light-conversion technologies [29,30]. As one of the most promising light conversion technologies, photocatalytic technology only needs the appropriate semiconductor photocatalyst and solar energy as energy input. In a typical photocatalytic procedure, the semiconductor photocatalysts can firstly absorb photons and be excited to generate hole-electron pairs under light irradiation. After separating and migrating to the surface of the photocatalysts, the two kinds of photo-generated charge carriers participate in redox reactions to produce free radicals and realize energy production or pollutant removal. However, the hole and electron can inevitably recombine in the bulk or on the surface of the photocatalysts, which is a disadvantageous factor [31]. In summary, the capacity of light absorption, the rate of photogenerated charge separation, migration and recombination are all influences on the photocatalytic process. The dominant factors can determine the photocatalytic efficiency of a given photocatalyst.

**Figure 1:**
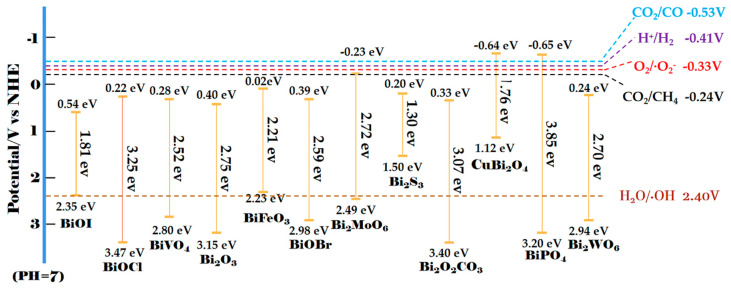
The bandgap structure of part of representative Bi-based semiconductors.

Research on semiconductor photocatalysts has attracted considerable attention since water splitting and hydrogen production on titanium dioxide (TiO_2_) were first reported by Fujishima et al. [32]. TiO_2_ is one of the most extensive studied semiconductors because of its chemical stability, non-toxicity, low cost, and good corrosion resistance [33,34]. Nevertheless, due to its wide bandgap (3.2 eV), TiO_2_ can only absorb ultraviolet light energy which constitutes about 4–5% of solar energy. The poor solar energy utilization capacity leads to the low quantum efficiency and the practical application of TiO_2_ is greatly limited [35,36]. Considering that visible light energy accounts for about 45% of sunlight energy [37], subsequently, in order to broaden the light-harvesting range, remarkable efforts have been devoted to explore visible light-activated semiconductors including CdSe, CdS, SiC, WO_3_, Fe_2_O_3_, Co_3_O_4_, g–C_3_N_4_, CdO, Ag_2_O et al. as photocatalysts, which possess a narrow band gap that is less than or equal to 3.0 eV. Recently, bismuth-based (Bi-based) semiconductors with advantages of non-toxicity, low cost and good thermal stability and as new and important visible light-activated semiconductors have become a research hotspot [20,38,39,40,41,42,43,44,45,46,47,48]. Bi-based semiconductors usually refer to the Bi^3+^ containing semiconductors because of the higher stability of Bi^3+^ than Bi^5+^. Various Bi-based semiconductors have been proved as excellent photocatalytic materials such as BiOX (X = Cl, Br, I) [49,50,51], BiVO_4_ [52], Bi_2_O_3_ [53], Bi_2_S_3_ [54], BiFeO_3_ [21], Bi_2_Sn_2_O_7_ [55], Bi_2_MoO_6_ [56], Bi_2_WO_6_ [24], CuBi_2_O_4_ [20], Bi_3_ClO_4_ [16], Bi_2_O_2_CO_3_ [23]. The bandgap structure of part of representative Bi-based semiconductors is summarized in Figure 1 [20,38,39,40,41,42,43,44,45,46,47,48]. As shown in Figure 1, the bandgap of the vast majority of Bi-based semiconductors is less than 3.0 eV except for BiPO_4_, BiOCl, Bi_2_O_2_CO_3_ and the like, which means that most Bi-based semiconductors can be excited by visible light. However, because of the valence band (VB) potential and the conduction band (CB) potential of Bi-based semiconductors with a narrow band gap not being positive and negative enough, the photogenerated holes and electrons do not have sufficient redox ability to drive the specific photocatalytic reactions, for example to produce free radicals including hydroxyl radical (OH) and superoxide radical (O_2_^−^), which is crucial for photocatalytic pollutant removal. Moreover, compared with wide-bandgap semiconductors, the excited electrons recombine with holes more easily and quickly in narrow-bandgap semiconductors. Among the Bi-based semiconductors shown in Figure 1, only BiPO_4_ has sufficient redox capacity to produce both OH and O_2_^−^, whereas BiPO_4_ has a larger bandgap, which is unfavorable for visible-light energy utilization. Consequently, two inherent limitations exist. One is that a given Bi-based semiconductor with a single component cannot have the strong redox ability and the good solar energy utilization capacity simultaneously. The other is that the recombination of the photogenerated electron-hole pairs inhibits the photocatalytic performance of a given Bi-based semiconductor with single component.

In recent years, tremendous attempts have been made to improve the visible-light absorption ability, light-harvesting efficiency, and photogenerated carriers’ separation of Bi-based semiconductors. Rongan He, Jiaguo Yu, and Wingkei Ho et al. have reviewed the strategies for enhancing the performance of bulk Bi-based semiconductors including component adjustment, morphology control, heterojunction construction, and surface modification [57]. Although the component adjustment, morphology control, and surface modification can improve the photocatalytic performance of bulk Bi-based semiconductors with a single component to some extent, the first inherent limitation is still not overcome. However, heterojunction construction, especially Z-scheme heterojunction construction, is considered a promising strategy to conquer the conflict between good solar energy utilization ability and excellent redox capacity for single-component Bi-based semiconductor. From the development history of the Z-scheme heterojunction, it can be divided into three generations: liquid-phase Z-scheme, all-solid-state Z-scheme and direct Z-scheme photocatalyst [58]. Among them, the third generation Z-scheme heterojunction, direct Z-scheme photocatalyst, possesses the largest application range, the fastest charge-carrier migration rate, and the lowest fabrication cost because, unlike the first two generations with the help of electron mediator to transfer charge carriers, its charge-carrier migration driving force is the internal electric field which is formed due to the charge redistribution upon the contact of semiconductor components. The comprehensive description of the basic principle of the Z-scheme heterojunction can be found in some published review articles [59,60].

The present mini review focuses on recent achievements in a number of synthesis methods of Bi-based direct Z-scheme heterojunction photocatalysts and different photocatalytic applications of Bi-based Z-scheme heterojunction photocatalysts, such as photocatalytic degradation and photoelectrocatalytic detection of environmental pollutants, water splitting, CO_2_ reduction, and air (NO_x_) purification. Finally, the challenges, prospects, and future directions for Bi-based Z-scheme heterojunction photocatalysts are presented.

## 2. Synthesis Methods of Bi-Based Direct Z-Scheme Photocatalysts

**Figure 2:**
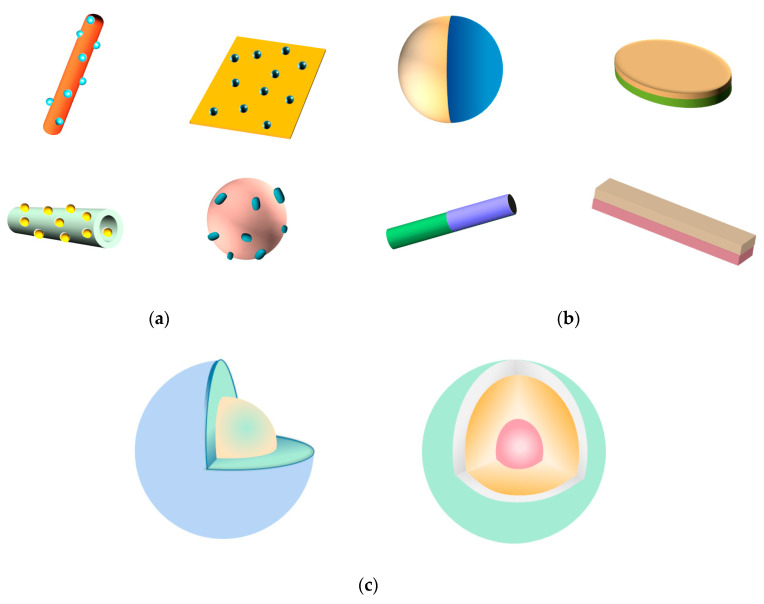
The schematic diagram of (**a**) surface decorated structure; (**b**) Janus structure; and (**c**) core-shell structure.

Different synthesis methods can obtain Bi-based direct Z-scheme photocatalysts with different interfacial properties, geometrical configurations (see Figure 2), morphology, and crystallinity and so on, which eventually affect the photocatalytic performance of the prepared Bi-based direct Z-scheme photocatalysts. It is widely known that both morphology (size, shape and dimensionality) and crystallinity have a substantial influence on the properties of semiconductor oxides and further on the photocatalytic performance of the direct Z-scheme photocatalysts that they make up. In order to optimize the performance of semiconductor components, a variety of synthesis methods have been developed to control the morphology and crystallinity of semiconductor components by adjusting the parameters of synthesis methods. For example, Yongfa Zhu et al. [61] prepared a series of Bi_2_MoO_6_ with different morphologies (from 2D nanosheet to 1D microrod structures) by adjusting the pH value of the reactant through a hydrothermal method. Similarly, uniform BiOCl hierarchical microspheres assembled by nanosheets with tunable thickness were synthesized via a simple solvothermal route [62]. Bi_2_O_3_ with different hierarchitectures were reported to be controllably synthesized by modulating the experimental conditions of the template-free method, such as VO_3_^−^ concentration, the reaction temperature, and the pH values [63].

However, for a direct Z-scheme photocatalyst, just optimizing the properties of its semiconductor components is not enough, the interfacial condition among the semiconductor components is crucial, considering the charge-carrier migration driving force of direct Z-scheme photocatalyst is related to the contact of semiconductor components. It is noteworthy that the stronger the interaction and intimate interface among the components of the Z-scheme heterojunction, the higher the efficiency of charge carrier separation and transfer [64]. Moreover, the geometrical configuration of direct Z-scheme photocatalyst is also important because it affects the contact mode of semiconductor components in Bi-based direct Z-scheme photocatalysts. In the surface decorated structure (Figure 2a), the components of the Bi-based Z-scheme heterojunction are all exposed to the reaction environment and can be excited by light and participate the redox reactions. However, excess amount of decorated component will inhibit the light absorption of other components, which can be improved by Janus structure (Figure 2b). In a core-shell structure (Figure 2c), the core component can be protected from light corrosion or undesired dissolution. Nevertheless, because of the protection, the core component cannot be excited by light and participate the redox reactions, which is not of benefit to the charge consumption and further charge carrier transfer between the core and shell component. Table 1 lists the advantages and shortcomings of extensively used synthesis methods of Bi-based direct Z-scheme photocatalysts including hydrothermal and solvothermal method, solid-state synthesis, deposition-precipitation method, cation exchange method, electrospinning method, self-assembly method, mechanical agitation method, and ultrasonic chemical method. The interfacial properties and geometrical configurations of the prepared Z-scheme heterojunction by these synthesis methods are also compared in Table 1 [65,66,67,68,69].

In the tables of Section 3, we summarize the representative examples of the aforementioned synthesis methods of Bi-based direct Z-scheme photocatalysts from recent related works.

## 3. Applications of Bi-Based Z-Scheme Photocatalysts

Due to the excellent redox capacity, good solar energy utilization ability, and efficient hole-electron separation ability, superior photocatalytic performance, Bi-based Z-scheme heterojunctions have been achieved in a wide range of applications, such as degradation of pollutants [70], water splitting [68], CO_2_ reduction [71], detection of environmental pollutants [72], removal of NO_x_ [73], etc. In this section, various photocatalytic applications of Bi-based Z-scheme photocatalysts are summarized and briefly presented.

### 3.1. Degradation of Pollutants in Water

**Figure 3:**
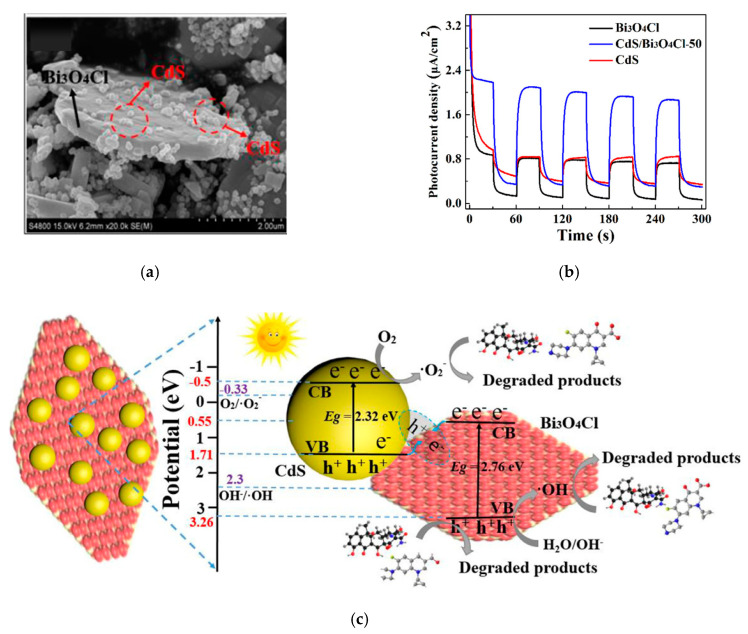
(**a**) Scanning electron microscope (SEM) image of CdS/Bi_3_O_4_Cl-50 heterostructure; (**b**) the transient photocurrent response of the bare CdS, Bi_3_O_4_Cl and Z-Scheme CdS/Bi_3_O_4_Cl-50 heterostructure; (**c**) possible photocatalytic mechanism of Z-scheme CdS/Bi_3_O_4_Cl photocatalyst for antibiotic treatment under visible light irradiation; CdS/Bi_3_O_4_Cl-50: mass ratio of Bi_3_O_4_Cl to CdS is 50. Reproduced with permission from [75]. Copyright Elsevier, 2018.

With the rapid development of the economy, large-scale industrialization and urbanization make environmental pollution a very serious problem, which not only hinders the sustainable development of society, but also threatens the life and safety of human beings [74]. Owing to the unique structure of a Bi-based Z-scheme photocatalyst, the redox potential of the heterojunction can be maximized, the solar energy can be almost utilized completely, and the photogenerated electrons and holes can be separated efficiently. The Bi-based Z-scheme photocatalyst is reported to be a promising photocatalyst for photocatalytic degradation of various environmental pollutants. Table 2 lists some of the latest and most representative research on degradation of pollutants by Bi-based Z-scheme photocatalyst. As shown in Table 2, the photocatalytic efficiency usually was expressed by the degradation rate of pollutants (%) or the kinetic constant k (min^−1^). It has been shown that compared with corresponding single Bi-based photocatalyst, the Z-scheme heterojunction greatly improves the photocatalytic degradation efficiency of pollutants. For example, Liu et al. [75] prepared Z-scheme Bi_3_O_4_Cl/CdS by the simple hydrothermal method. Briefly, CdS nanospheres and Bi_3_O_4_Cl nanosheets were synthesized firstly by the hydrothermal method. Then, with the assist of polyvinyl pyrrolidone (PVP), Z-scheme Bi_3_O_4_Cl/CdS was synthesized by a facile surfactant-free hydrothermal treatment. The results showed that the CdS nanospheres are successfully and uniformly loaded on the surface of Bi_3_O_4_Cl nanosheets forming a surface-decorated heterostructure and an efficiently intimate heterojunction interface (see Figure 3a). The heterojunction presents an obviously enhanced absorption in the visible region compared to pure Bi_3_O_4_Cl. The significant improvement of charge transfer and separation of the composite was proved by photocurrent (see Figure 3b) and electrochemical impedance spectra (EIS) measurements. Under visible light illumination, Bi_3_O_4_Cl/CdS composite displays higher photocatalytic activity towards the ciprofloxacin (CIP) and tetracycline (TC) degradation than pure Bi_3_O_4_Cl, which is ascribed to the direct Z-scheme mechanism (See Figure 3c). The direct Z-scheme mechanism was proved by active species trapping experiments and electron spin resonance (ESR) technology. As shown in Figure 3c, the redox potential of the Z-scheme Bi_3_O_4_Cl/CdS heterojunction can be maximized sufficiently to produce OH (2.40 V vs. NHE (normal hydrogen electrode)) and O_2_^−^ (−0.33 V vs. NHE).

Zeng et al. [83] fabricated ternary Z-scheme heterojunction (meso-tetra (4–carboxyphenyl) porphyrin (TCPP)/reduced graphene oxide (rGO)/Bi_2_WO_6_ (BWO)) via an ultrasonic chemical method. Firstly, rGO/BWO was prepared by the hydrothermal method. Then, rGO/BWO was added to absolute ethanol and ultrasonicated for dispersal. TCPP was added to the above suspension and kept in ultra-sonication then stirred until the solvent was completely volatilized. Finally, the product was dried to obtain the TCPP/rGO/BWO. During the synthetic process, the carboxylic groups of TCPP make it combine with the BWO tightly. The introduction of rGO further improves the photocatalytic performance of the composite because of its π-π structure for efficient contaminants adsorption, great photo-response property for expanding visible-light response range, and high electron mobility for promoting charge transfer and separation. The structure of the prepared TCPP/rGO/BWO is a surface-decorated heterostructure, which was proved by scanning electron microscopy (SEM) results. The TCPP0.25/rGO/BWO has the best photocatalytic performance for the degradation of tetracycline (TC) compared with BWO, rGO/BWO, TCPP/BWO, and other TCPP/rGO/BWO composites with different TCPP contents. The results of trapping experiments and ESR analysis indicated that the h^+^ and O_2_^−^ are the major contributors for the TC decomposition in the TCPP0.25/rGO/BWO system. The O_2_^−^ can only be produced via reducing O_2_ by the electrons at the lowest unoccupied molecular orbital (LUMO) (−0.60 eV) of TCPP which is above the energy level of the O_2_/·O_2_^−^ (−0.33V vs. NHE). Therefore, a Z-scheme mechanism is proposed to elucidate the charge transfer process in the TCPP0.25/rGO/BWO system to ensure the efficient charge separation and sufficient redox potential.

### 3.2. Water Splitting

Solar-water splitting can convert solar energy into clean, carbon-neutral and storable chemical energy (hydrogen fuel) without using fossil fuels and causing carbon emissions. Therefore, solar-water splitting has attracted much attention [95]. 

**Figure 4:**
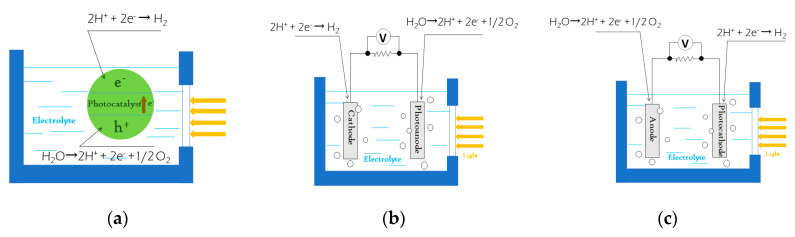
Schematic diagram of photocatalytic hydrogen production principle of photocatalyst dispersed in electrolyte (**a**), schematic diagram of hydrogen production principle in a photoelectrocatalytic system composed of a n-type semiconductor as photoanode (**b**), and a p-type semiconductor as photocathode (**c**), respectively.

The photocatalytic (PC) and photoelectrocatalytic (PEC) methods are two simple, efficient, low-cost and environmentally benign means for achieving solar-water splitting. As shown in Figure 4a, during the photocatalytic process, the photocatalysts that are highly dispersed in solution can be excited by solar light and produce electron-hole pairs which further participate in the redox reactions to split water to hydrogen (H_2_) and oxygen (O_2_). Unlike in the PC system where photocatalysts are dispersed in the solution, in the PEC system the photocatalysts should be attached on the working electrode to construct the PEC system and in order to apply an external bias. Although the external bias is beneficial to promote the charge separation and reaction kinetics and attached photocatalysts are easy to reuse, the specific surface area and the photocatalytic active sites of attached photocatalysts on the electrode is far less than dispersed photocatalysts in the solution. In the PEC water splitting system, at least one photoelectrode should be required for utilizing solar energy. Depending on the type of photocatalyst on the photoelectrode, PEC water splitting system has different hydrogen production principles. As displayed in Figure 4b, when the photoelectrode consisting of n-type semiconductor photocatalyst as photoanode is excited by solar energy, the photogenerated holes will oxidise water molecule to hydrogen ions and O_2_, see Equation (1). The photogenerated electrons will transfer to the counter electrode (cathode) via an external circuit to reduce the hydrogen ions to hydrogen, see Equation (2) [96]. By contrast, when the photocatalyst is p-type semiconductor and as photocathode, the H_2_ and O_2_ will evolve at the surface of photocathode (see Equation (2)) and anode (see Equation (1)), respectively (see Figure 4c). The two basic semi-reactions and their overall reaction (Equation (3)) are as follows:

(1) $$anode:\;2H_{2}O+4h^{+}\to O_{2}+4H^{+}\;(E^{0}{}_{OX}=+1.23\;V\;at\;pH=7)$$

(2) $$cathode:\;2H^{+}+2e^{-}\to H_{2}\;(E^{0}{}_{RED}=0\;V\;at\;pH=0)$$

(3) $$Overall:\;2H_{2}O\to 2H_{2}+O_{2}$$

According to the aforementioned semi-reactions, in order to realize overall water splitting, the selected ideal photocatalyst should satisfy the requirements that the CB edge potential of the photoelectrode semiconductor should be above the energy level of the H^+^/H_2_ (0 V vs. NHE at pH = 0) and the VB edge potential should be below the energy level of the O_2_/H_2_O (1.23 V vs. NHE at pH = 0) [97]. The Z-scheme heterojunction provides a promising way to overcome the thermodynamic energy barrier for solar-water splitting by combining a narrow bandgap semiconductor which ensures the maximized utilization solar energy and one or more paired semiconductors with appropriate energy band structure to broaden the redox range of the photocatalyst composite. Bi-based semiconductors are promising candidates for a Z-scheme heterojunction attributed to their unique energy band structure and other attractive advantages. Table 3 summarizes the latest development of Bi-based Z-scheme photocatalysts applied for solar-water splitting. The Bi-based Z-scheme photocatalyst and its synthesis method, the conditions of the solar-water splitting process, the utilization of co-catalyst, the products and yields, and apparent quantum yield (AQY) of all the examples are presented in Table 3. From the data of Table 3, it is seen that Bi-based Z-scheme photocatalysts exhibit promising performance for solar-water splitting. Most research using Bi-based Z-scheme photocatalysts can obtain good H_2_ evolution yield. Chou et al. [98] made SnS_2_ self-growth on the BiPO_4_ nanosheets to form three dimensions (3D) flower heterogeneous composite by a multi-step solvothermal method. According to the radical-trapping experiment results and band structure analysis, a Z-scheme heterojunction was formed between SnS_2_ and BiPO_4_. Under visible light, the formed Z-scheme heterojunction showed the highest H_2_ evolution rate of 303 μmol h^−1^ g^−1^, which is about 1.43 and 2.01 times higher than that of pure SnS_2_ and pure BiPO_4_, respectively.

**Figure 5:**
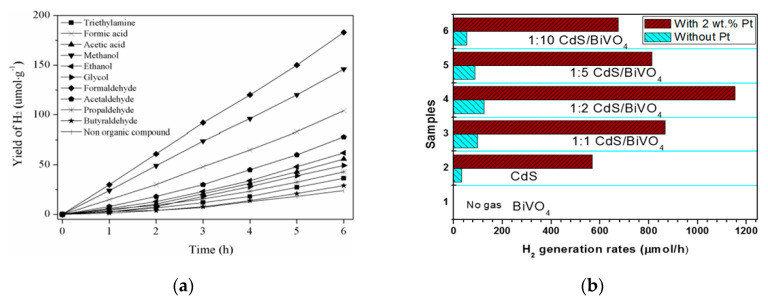
(**a**) Effects of different organic compounds on hydrogen production in ZnIn_2_S_4_/RGO/BiVO_4_ photocatalyst. Reproduced with permission from [106]. Copyright Elsevier, 2019; (**b**) Photocatalytic H_2_ generation rates of different mass ratios CdS/BiVO_4_ in lactic acid solution with or without 2 wt.% Pt under visible light irradiation. Reproduced with permission from [103]. Copyright Elsevier, 2017.

Due to the high energy barrier of its four-electron transfer process, the O_2_-evolution half reaction in overall solar-water splitting is much more challenging than the H_2_-evolution half reaction, which dramatically suppresses the efficiency of the overall solar-water splitting. Usually, most semiconductor photocatalysts have low activity for O_2_ evolution [109]. Besides the overall solar-water splitting, H_2_-evolution half reaction using a sacrificial agent is another efficient way to harvest and convert solar energy to H_2_. The sacrificial agent plays a significant role in the H_2_-evolution half reaction. Suitable sacrificial reagents can improve the H_2_-evolution efficiency remarkably by scavenging the photogenerated holes to reduce the charge carrier recombination significantly. Zhu et al. investigated the effect of the different organic compounds on the hydrogen production rate of ZnIn_2_S_4_/RGO/BiVO_4_ [106]. As shown in Figure 5a, in the presence of organic compounds such as formaldehyde, methanol, formic acid, acetaldehyde and ethanol and so on, the hydrogen production rate has been promoted to a different extent. Organic compounds including alcohols (for example methanol [99]), aldehydes (for example formaldehyde [106]), organic acids (for example lactic acid [103]) have been extensively used as efficient hole scavengers for H_2_ production in Bi-based Z-scheme photocatalysts systems.

Furthermore, owing to the sufficient redox ability of the Bi-based Z-scheme photocatalyst, even refractory organic pollutants can also be successfully used as hole scavengers to achieve simultaneous H_2_ generation and pollutant degradation. Very recently, Liu et al. constructed a Cu_2_O/BiVO_4_ Z-scheme heterojunction by using reduced graphene oxide (rGO) as an adhesive via a two-step solvothermal method. This Bi-based Z-scheme photocatalyst exhibits excellent photocatalytic performance on simultaneous tetracycline (TC) degradation and H_2_ production under visible light irradiation [108].

Moreover, the introduction of a suitable co-catalyst can further improve the solar-water splitting efficiency of a Bi-based Z-scheme photocatalyst. The reason for this improvement may be attributed to the fact that co-catalysts can efficiently collect photogenerated carriers and catalyze H_2_ or O_2_ evolution as well as improve the stability of photocatalysts by suppressing photocorrosion [110]. Usually, noble metals and especially Pt have been extensively used as co-catalyst for solar-water splitting [111]. For example, Zhou et al. [103] prepared hierarchical CdS/BiVO_4_ hybrid composed of CdS nanoparticles decorated on BiVO_4_ nanowires (NWs) by the solvothermal method. The strongly chemical interaction between CdS and BiVO_4_ was confirmed by XPS (X-ray photoelectron spectroscopy) results. As illustrated in Figure 5b, under visible light irradiation and in a lactic acid electrolyte, no hydrogen generation was observed on pure BiVO_4_ even loaded with Pt as co-catalyst, attributed to the CB edge potential of pure BiVO_4_ being lower than the energy level of H^+^/H_2_. The bare CdS showed a weak hydrogen production capacity. After forming a CdS/BiVO_4_ Z-scheme heterojunction, the hydrogen production capacity was enhanced, whereas the improvement of photocatalytic H_2_ activity was by orders of magnitude after loading Pt. At an optimized condition, CdS (50 wt.%) /BiVO_4_ NWs with loading 2 wt.% Pt exhibited the fastest photocatalytic H_2_ generation rate which is 9.30 times of that of CdS (50 wt.%)/BiVO_4_ NWs without loading Pt co-catalyst. Therefore, due to their low overpotential for H_2_ evolution and excellent electron-accepting capacity, using noble metal as a co-catalyst is an effective method to improve the photocatalytic hydrogen evolution, which is conducive to the electron generated by the photocatalyst to transfer to the noble metal to catalyze H_2_ evolution.

**Figure 6:**
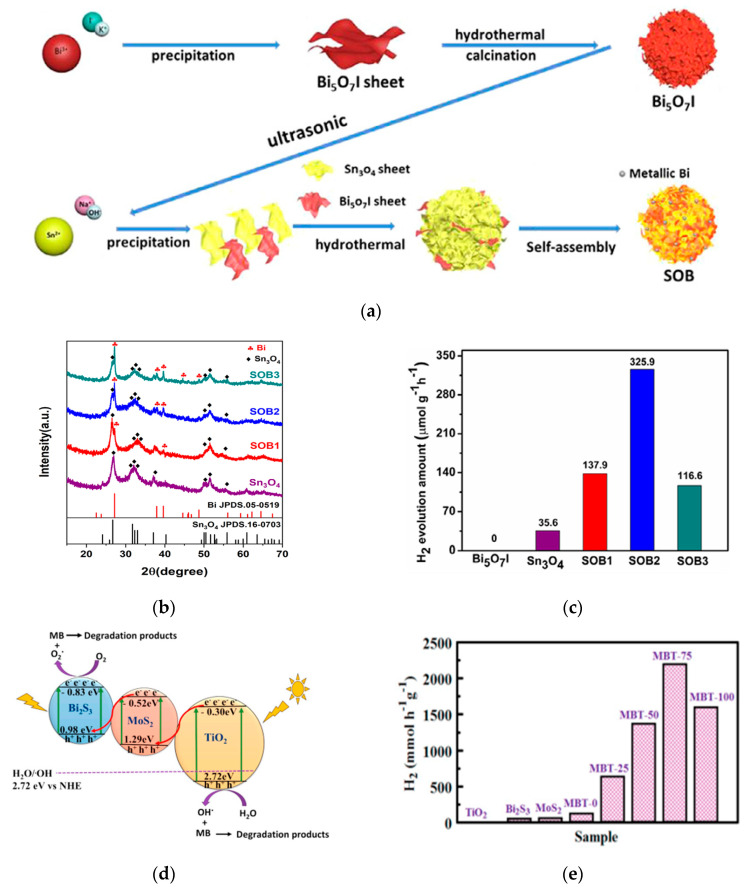
(**a**) The schematic diagram of Bi/Bi_5_O_7_I/Sn_3_O_4_ preparation process; (**b**) X-ray diffraction (XRD) patterns of Sn_3_O_4_ and Bi/Bi_5_O_7_I/Sn_3_O_4_ complexes (Bi/Bi_5_O_7_I/Sn_3_O_4_ samples doped 15 mg, 30 mg, and 45 mg Bi_5_O_7_I were labeled as SOB1, SOB2 and SOB3, respectively); (**c**) Average hydrogen production of Sn_3_O_4_ and SOB heterojunction within 5 h. Reproduced with permission from [99]. Copyright Elsevier, 2020; (**d**) Mechanism diagram of Z-scheme Bi_2_S_3_/MoS_2_/TiO_2_ heterojunction; (**e**) Comparison of H_2_ production performance of Bi_2_S_3_/MoS_2_/TiO_2_ (MBT) samples with different TiO_2_ contents. Reproduced with permission from [104]. Copyright Elsevier, 2020.

However, the high price and scarcity of noble metals limit their large-scale practical application in photocatalytic H_2_ generation as co-catalysts. In recent years, many efforts were devoted to develop co-catalysts composed of abundant and inexpensive elements to assist Bi-based Z-scheme photocatalyst for solar-water splitting. Recently, Xu et al. [99] prepared Bi/Bi_5_O_7_I/Sn_3_O_4_ by hydrothermal method (See Figure 6a). The band structure of Bi_5_O_7_I matches well with that of Sn_3_O_4_ to constitute a direct Z-scheme heterojunction. Metallic Bi which is evenly covered on the surface of Z-scheme Bi_5_O_7_I/Sn_3_O_4_ photocatalyst and comes from the reduction reaction between Sn_3_O_4_ and Bi_5_O_7_I during the synthesis process was demonstrated to be a good substitute for noble metals as co-catalyst to further improve the H_2_ generation and extend the light absorption range. The X-ray diffraction (XRD) results (see Figure 6b) confirmed the presence of Bi metal. As shown in Figure 6b, as increasing the amount of Bi_5_O_7_I, the characteristic peak of Bi gradually appears and increases. The photocatalytic hydrogen production experiments under visible light irradiation demonstrated that the highest hydrogen evolution of this Z-scheme catalyst reached 325.9 μmol h^−1^·g^−1^ without any noble metal co-catalyst and exceeded the rate on pure Sn_3_O_4_ by 5 times (See Figure 6c).

Great processes have been made to construct double Z-scheme heterojunction structures to make full use of solar energy to apply in water splitting. Drmosh et al. [104] prepared Bi_2_S_3_/MoS_2_/TiO_2_ (MBT) by a facile microwave-assisted hydrothermal method. The double Z-scheme heterojunction structure was constructed due to the matched band structure among Bi_2_S_3_ nanorods, MoS_2_ nanometer sheets and TiO_2_ nanotubes. As exhibited in Figure 6d, photogenerated electrons in the CB of TiO_2_ can recombine with photogenerated holes in the VB of MoS_2_, and photogenerated electrons in the CB of MoS_2_ can recombine with photogenerated holes in the VB of Bi_2_S_3_, preserving the photogenerated electrons and holes with the strongest reduction and oxidization power. The constructed direct double Z-scheme heterojunction extends the light-harvesting capability, couples the respective advantages of each component, and efficiently separates photogenerated electron-hole pairs. The optimized Bi_2_S_3_/MoS_2_/TiO_2_ nanocomposites presented a high photocatalytic H_2_-production rate of 2195 μmol h^−1^·g^−1^ under the sunlight irradiation, even in the absence of any noble-metal cocatalyst (See Figure 6e).

### 3.3. CO_2_ Reduction

Nowadays, excessive carbon dioxide (CO_2_) emission is a key reason for global warming. Proposing environmentally friendly and efficient strategies to dramatically reduce atmospheric CO_2_ is essential and urgent. Photocatalysis seems a propitious and appealing strategy because it can directly make use of inexhaustible solar energy to convert CO_2_ in the atmosphere into hydrocarbons such as CH_4_, HCOOH, CO, CH_2_O and CH_3_O (Equations (4)–(9)) [33,112], alleviating the greenhouse effect and energy crisis simultaneously. The product species of photocatalytic CO_2_ reduction depend on the relationship between the CB edge potential of the photocatalyst and the reduction potentials of the desired CO_2_ reduced product. Since CO_2_ is well known as one of the most thermodynamically stable chemical species [113,114], to satisfy thermodynamic requirements, the more negative the CB edge potential of the photocatalyst, the higher the possibility to drive the CO_2_ reduction process. On the other hand, the photocatalytic CO_2_ reduction system ultimately needs the use of water as the electron source (Equation (10)); a semiconductor photocatalyst has to satisfy band edge potentials that straddle both the water oxidation and CO_2_ reduction potentials.

(4) $$CO_{2}+8H^{+}+8e^{-}\to CH_{4}+2H_{2}O\;E^{0}=-0.24\;V\;vs\;NHE\;at\;pH=7\;$$

(5) $$CO_{2}+6H^{+}+6e^{-}\to CH_{3}OH+H_{2}O\;E^{0}=-0.38\;V\;vs\;NHE\;at\;pH=7\;$$

(6) $$CO_{2}+4H^{+}+4e^{-}\to HCHO+H_{2}O\;E^{0}=-0.48\;V\;vs\;NHE\;at\;pH=7$$

(7) $$CO_{2}+2H^{+}+2e^{-}\to CO+H_{2}O\;E^{0}=-0.53\;V\;vs\;NHE\;at\;pH=7\;$$

(8) $$CO_{2}+2H^{+}+2e^{-}\to HCOOH\;E^{0}=-0.61\;V\;vs\;NHE\;at\;pH=7$$

(9) $$CO_{2}+e^{-}\to \cdot CO_{2}^{-}\;E^{0}=-1.90\;V\;vs\;NHE\;at\;pH=7\;$$

(10) $$H_{2}O+2h^{+}\to 2H^{+}+1/2O_{2}\;E^{0}=+0.82\;V\;vs\;NHE\;at\;pH=7$$

The photocatalytic CO_2_ reduction reaction usually includes four main steps as follows: (1) CO_2_ adsorption process on the active sites of photocatalyst; (2) absorption of sufficient incident photon energy by the photocatalyst to generate electron-hole pairs; (3) charge separation and migration to the surface of the photocatalyst; (4) surface reactions for CO_2_ reduction and products desorption. In order to efficiently photocatalytically reduce CO_2_, the photocatalyst should possess all the features to successfully achieve each above step: including strong CO_2_ selective adsorption capacity, broad light response range, high charge separation efficiency, and sufficiently strong redox activity. Due to the aforementioned last three virtues, Z-scheme photocatalytic systems have been extensively investigated and applied for CO_2_ reduction in recent years. Based on the prominent photocatalytic activity nature of bismuth semiconductor, the representative progresses of Bi-based Z-scheme photocatalytic systems for the application of CO_2_ reduction are summarized in Table 4.

The CO_2_ adsorption process can be improved by using the photocatalyst with large surface area. Modulating the morphology is an efficient way to obtain the photocatalyst with large surface area. Jung’s group reported Z-scheme BiVO_4_/carbon-coated Cu_2_O (BVO/C/Cu_2_O) nanowire arrays (NWAs) with a three-dimensional (3D) structure for efficient photoconversion of CO_2_ to CO and CH_4_ [124]. The highest CO formation rate on this BVO/C/Cu_2_O NWAs reached 3.01 μmol h^−1^·g^−1^, which is about 9.4 and 4.7 times on Cu_2_O mesh and Cu_2_O NWAs, respectively. The dramatically enhanced photocatalytic activity was ascribed to the construction of a Z-scheme on a 3D NWAs structure. Enlarged surface area and enhanced charge-carrier transfer of 3D NWAs structure was evidenced by the electrochemical surface area method and photocurrent experiment results, respectively. Owing to the large surface area, enhanced charge-transport property, and light scattering or reflecting effect of the 3D NWAs structure, the combination of unique 3D morphology with a Z-scheme charge flow is not only beneficial for the efficient charge separation and transfer [125], but also favorable for facilitating the light absorption and CO_2_ adsorption by providing ample active sites [126,127]. Moreover, the thermodynamic feasibility of the photocatalytic reduction of CO_2_ and water oxidation on this BVO/C/Cu_2_O NWAs was verified by the band edge configuration via ultraviolet photoelectron spectroscopy. The Z-scheme charge-transfer mechanism was confirmed by investigating the energy level of the photoinduced hole via a photoluminescence (PL) experiment using coumarin as a probe molecule in water.

Apart from morphology modulation of the photocatalyst, introducing a co-catalyst in the Bi-based Z-scheme system to improve the photocatalytic CO_2_ reduction process is another efficient way. Recently, Jo and coworkers [122] constructed a Z-scheme Bi_2_WO_6_/rGO/g–C_3_N_4_ (BWO/rGO/CN) for photocatalytic CO_2_ reduction. Therein, rGO with excellent conductivity and large specific area was used as a co-catalyst for the Z-scheme photocatalyst to not only facilitate charge-carrier migration in Z-scheme mode, but also benefit the CO_2_ adsorption and electron capture by establishing the unique π-π conjugation interaction and providing abundant active sites and further promoting the CO_2_ photoreduction. In addition, attributed to forming large intimate interfaces, the 2D/2D/2D configuration of BWO/rGO/CN possesses strong light absorption in the visible region and strong electron shuttling at the interfaces hindering the direct recombination of charge carriers. As a consequence, the optimized Z-scheme BWO/rGO/CN displayed a remarkable photocatalytic performance for not only CO_2_ reduction but also water splitting. The AQY of 0.75% at 400 nm was higher than the other state-of-the art CO_2_ photoreduction catalyst system. 

**Figure 7:**
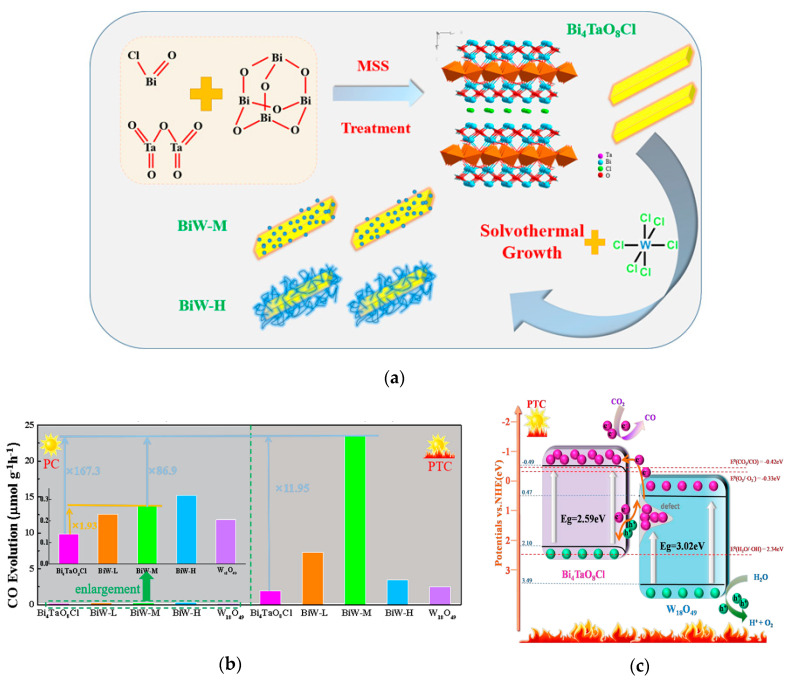
(**a**) The preparation process diagram of Bi_4_TaO_8_Cl/W_18_O_49_ heterojunction; (**b**) the amount of CO generated over different samples in the process of CO_2_ reduction under photocatalysis (PC, 298 K) and photothermocatalysis (PTC, 393 K); BiW-L, BiW-M, BiW-H mean the samples synthesized by adding the low, middle, and high content of WCl_6_ in the synthesis solution, respectively. (**c**) photothermal catalysis schematic diagram of Bi_4_TaO_8_Cl/W_18_O_49_ heterojunction. Reproduced with permission from [120]. Copyright Elsevier, 2020.

Moreover, integrating other techniques with photocatalytic CO_2_ reduction over a direct Z-scheme system will obtain unexpected results. The recently reported photothermal synergic enhancement of photocatalytic CO_2_ reduction performance of a direct Z-scheme Bi_4_TaO_8_Cl/W_18_O_49_ (BiW) system is an interesting example of the integration of the photocatalytic process with external heating [120]. The direct Z-scheme heterojunction was successfully fabricated by growing W_18_O_49_ nanostructures on the surface of a Bi_4_TaO_8_Cl nanosheet (See Figure 7a). The well matched energy band of these two semiconductors makes the constructed Z-scheme heterojunction a promising photocatalyst for CO_2_ reduction and H_2_O oxidation under visible light. Under photothermal conditions, the CO yield of the optimal BiW was increased surprisingly by 87 times over photocatalytic conditions (see Figure 7b). Interestingly, after light irradiation, long-lasting catalytic reduction of CO_2_ in the dark was observed. The possible reasons for these experimental results are that external heating enhances the Z-scheme behavior of the BiW heterostructure by helping electrons at electron traps detrap to the surface of photocatalyst to increase the efficiency of electron utilization and promote the CO_2_ reduction reaction, at the same time, decreasing the activation energy of lattice oxygen to promote oxidation reactions at the other reaction sites. The photoexcited electrons stored at the oxygen vacancy defects of W_18_O_49_ can be released and excited to the conduction band of Bi_4_TaO_8_Cl by heating to reduce CO_2_ and produce considerable CO in the dark after light irradiation (See Figure 7c). The design of this photothermal catalyst provides a novel and promising method for using solar energy to catalyze the reduction of CO_2_ to fuels.

From Table 4, the main product of CO_2_ photoreduction in most Bi-based Z-scheme systems are CO and CH_4_. By comparing all the reaction equations of products from CO_2_ reduction (Equations (4)–(9)), the redox potential E^0^ of CO_2_/CH_4_ (−0.24 V vs. NHE) is the least negative value, which means the reaction of producing CH_4_ is one in which a reaction occurs most easily from a thermodynamic viewpoint. The CO formation through simple two-electron reduction process of CO_2_ is easier to achieve than the other products’ generation through a multiple-electron reduction process of CO_2_. In order to photoreduce CO_2_ to obtain more carbonaceous products via more difficult multi-electron-transfer pathways, further exploration is still needed such as with the help of a noble metal cocatalyst. Meanwhile, water usually serves as an electron donor during CO_2_ photoreduction process. In ideal conditions, H_2_O should be oxidized into protons and O_2_ (Equation (10)). However, the generated protons actually can further react with photoexcited electrons to produce H_2_ (Equation (11)). At the same time, O_2_ also can react with photoexcited electrons (Equation (12)). These reactions form competitive relationship with CO_2_ photoreduction (Equations (4)–(9)), which makes the electron-transfer process of CO_2_ photoreduction more complicate [128,129]. Therefore, to understand in depth and illuminate the actual and complicated mechanism of photocatalytic CO_2_ reduction is also crucial.

(11) $$2H^{+}+2e^{-}\to H_{2}\;E^{0}=-0.42\;V\;vs\;NHE\;at\;pH=7\;$$

(12) $$O_{2}+e^{-}\to \cdot O_{2}^{-}\;E_{0}=-0.33\;V\;vs\;NHE\;at\;pH=7$$

### 3.4. Removal of Gas Phase Pollutants and Other Applications

In recent years, the emission of nitrogen oxides (NO_x_) from human activities into the atmosphere has become one of the major environmental problems because the great harm of NO_x_ to human health, climate, and agriculture [130]. In addition, NO_x_ mainly composed of NO and NO_2_ can produce secondary aerosols and cause more serious environmental problems, such as acid rain, haze, photochemical smog, PM 2.5 (fine particulate matter with less than 2.5 µm diameter), and ozone accumulation, etc. [131,132]. Among various methods for NO_x_ removal, such as physical adsorption [133], biofiltration [134], and thermal catalytic reduction [135], photocatalysis, as a green chemical method, is considered to be a promising strategy due to its high efficiency and low cost. It has been reported that Bi-based Z-scheme heterojunction systems exhibit high photocatalytic activity for pollutant removal including NO_x_ removal because of the abundant active radicals with strong redox ability in these systems.

Zhu et al. [136] prepared a two-dimensional/two-dimensional (2D/2D) direct Z-scheme photocatalyst Bi_2_O_2_CO_3_/Bi_4_O_5_Br_2_ (BOC/BOB) by a simple one-pot hydrothermal method. The X-band electron spin resonance (ESR) spectra of O_2_^−^ and OH radicals and the Fermi level calculations strongly substantiated the direct Z-scheme charge separation mechanism of 2D/2D BOC/BOB. Under simulated solar light illumination, the optimizing BOC/BOB exhibited a significantly higher photocatalytic activity (53.2%) for NO_x_ removal than that of single-phase BOC (20.4%) and BOB (37.9%). Such improved photocatalytic activity was mainly attributed to the enhanced charge carriers’ separation efficiency and strong redox activity of remaining photogenerated charge carriers of a direct Z-scheme mode at the BOC/BOB interface. As revealed by the results of trapping experiments and ESR tests, both O_2_^−^ and·OH were the major active species for photocatalytic NO_x_ removal. Similarly, direct Z-scheme Bi_2_MoO_6_/ZnIn_2_S_4_ composite semiconductor photocatalysts were successfully constructed by a facile wet impregnation method and applied for the oxidative removal of NO with H_2_O_2_ solution injected and under visible light [73]. The optimal Bi_2_MoO_6_/ZnIn_2_S_4_ exhibited superior photocatalytic activity for NO removal, and the removal efficiency reached 84.94% in 80 min, attributed to the low rate of recombination of photogenerated charge carriers in the direct Z-scheme charge transfer mode.

Volatile organic compounds (VOCs), such as alcohols, aldehydes, ketones, alkenes, and aromatic compounds, are known to cause enormous harm to human health [137]. However, highly effective VOC elimination still remains a challenge. Photocatalytic degradation is found to be an attractive and promising technique for the abatement of VOC because of its mild operation conditions (room temperature and atmospheric pressure), highly effective and thorough degradation, and good solar energy utilization capacity. Photocatalyst is one of the keys of this technique. Z-scheme photocatalysts with high specific surface area have been accepted as rational photocatalysts for photocatalytic degradation of VOCs. For example, it was shown that direct Z-scheme BiVO_4_/g-C_3_N_4_ with coral-like structure achieved efficient mineralization of toluene under visible light illumination [138]. Brunauer–Emmett–Teller (BET) measurement provided evidence for the high specific surface area of the coral-like structure which provides more active sites for the photocatalytic oxidization toluene. According to the results of the ESR and terephthalic acid photoluminescence (TA-PL), the enhanced photocatalytic performance of BiVO_4_/g–C_3_N_4_ was attributed to a direct Z-scheme migration. The degradation rate constant of the optimal BiVO_4_/g–C_3_N_4_ for toluene degradation reached 0.138 h^−1^.

**Figure 8:**
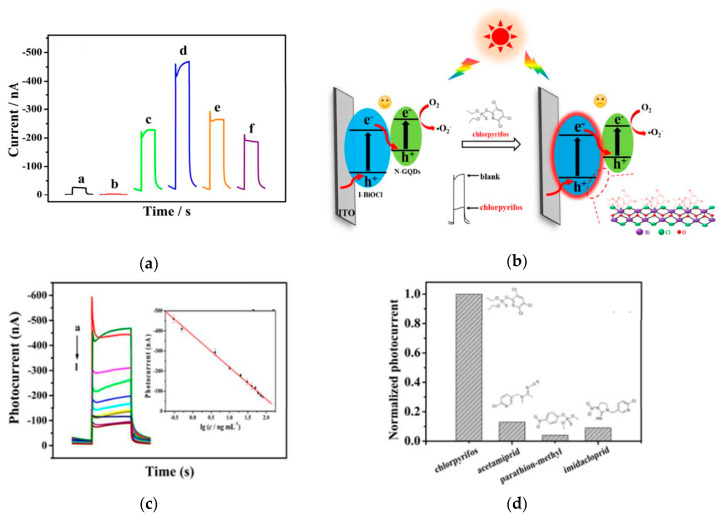
(**a**) Transient photocurrent of different photocatalysts (a: BiOCl, b: BiOI, c: I–BiOCl, d–f: I–BiOCl/N–GQDs-*x* (*x*: the volume of nitrogen-doped graphene quantum dots (N–GQDs), *x* = 0.5, 1.0, 1.5)); (**b**) schematic diagram of the “signal-off” cathodic PEC sensor constructed by I–BiOCl/N–GQDs for selective detection of chlorpyrifos; (**c**) Photocurrent response of I–BiOCl/N–GQDs-0.5 in the presence of 0 ng·mL^−1^, 0.3 ng·mL^−1^, 0.5 ng·mL^−1^, 4 ng·mL^−1^, 10 ng·mL^−1^, 20 ng·mL^−1^, 30 ng·mL^−1^, 40 ng·mL^−1^, 50 ng·mL^−1^, 60 ng·mL^−1^, 70 ng·mL^−1^, 80 ng·mL^−1^ chlorpyrifos (from a to l); Insert is the corresponding linear relationship between the log *C*_chlorpyrifos_ and photocurrent. (**d**) PEC response of I–BiOCl/N–GQDs-0.5 to chlorpyrifos and other foreign impurities. Reprinted with permission from [139]. Copyright ACS, 2018.

As a newly developed sensing technique, photoelectrochemical (PEC) sensors have attracted tremendous attention. Such PEC sensors combine the advantages of optical detection and electrochemical detection and improve their inherent defects at the same time. Owing to a detached excitation system and detection system, the PEC has high sensitivity, rapid measurement speed, and a low background signal. Additionally, in comparison to optical detection methods with costly optical imaging system and complicated image analysis software, the cost of PEC with a simple electrochemical detection system is much lower. From the sensing mechanism of PEC, the semiconductor as photoelectrode which is excited by light to generate hole-electron pairs and further produce photocurrent signal with the separation and migration of charge carriers is the core component of PEC. However, fast recombination of photogenerated electrons and holes as one of the inherent limitations of semiconductors is a big challenge for PEC sensors. The Z-scheme charge-carrier migration mode is beneficial to the charge separation and migration as well as improving the photoelectric conversion and signal generation efficiency of PEC. For instance, a Z-scheme iodine doped BiOCl/nitrogen-doped graphene quantum dots (I–BiOCl/N–GQDs) heterojunction was prepared by a one-pot precipitation method at room temperature [139]. Such a Z-scheme I–BiOCl/N–GQDs heterojunction was used as a photoelectrode to construct a “signal-off” cathodic PEC sensor for the selective detection of chlorpyrifos. The optimal I–BiOCl/N–GQDs composite exhibited the highest photocurrent signal (See Figure 8a) indicating excellent spatial separation efficiency of charge carriers which was evidenced by PL and EIS results. The Z-scheme charge carrier transfer pathway and the enhanced light harvesting can be used to explain the improved PEC performance. Figure 8b displays the PEC detection mechanism of chlorpyrifos. As shown in Figure 8b, in the presence of chlorpyrifos in the solution, the S and N atoms of chlorpyrifos will bind with the Bi(III) to form the bismuth-chlorpyrifos complex on the surface of I–BiOCl/N–GQDs, which will decrease the photocurrent signal because of the steric hindrance effect to achieve detection of chlorpyrifos. As shown in Figure 8c,d, this PEC sensor presented a wide linear detection range (0.3–80 ng·mL^−1^), considerably low detection limit (0.01 ng·mL^−1^), and good selectivity toward chlorpyrifos. Nevertheless, there is a lack of evidence for verifying the Z-scheme charge carrier transfer pathways in this work.

## 4. Conclusions and Perspectives

As one of the most promising light-conversion technologies, photocatalysis can ease the problem of energy and environmental pollution in the future by using appropriate semiconductor as photocatalysts and solar energy as energy input to drive photocatalytic reactions. Photocatalysts are a crucial factor which determine the performance of this technique. Bi-based semiconductors with advantages of non-toxicity, low cost and good thermal stability and as a new and important visible light-activated semiconductor have attracted great attention and become a research hotspot. Recently, great progress has been made in improving the visible-light absorption ability, light-harvesting efficiency, and photogenerated carriers’ separation of Bi-based semiconductors. However, for a single-component Bi-based semiconductor, it is hard to simultaneously satisfy strong redox ability for a specific photocatalytic reaction and the good solar energy utilization capacity. Z-scheme heterojunction construction is considered a promising strategy to conquer this conflict via combining a narrow bandgap semiconductor and one or more paired semiconductor with appropriate energy band structure to broad the light response range and enhance the redox ability of photocatalyst composite. Bi-based Z-scheme photocatalysts exhibit good performance in various photocatalytic applications involving energy production, for example, water splitting, environmental remediation such as CO_2_ reduction, NO_x_ removal, pollutants degradation, as well as pollutant detection, for example, construction of PEC sensors.

However, the study of the Bi-based Z-scheme heterojunction is still in its infancy and has many challenges and problems: (1) selecting two or more semiconductors with good matched band structure is the prerequisite for the successful preparation of direct Z-scheme photocatalysts. Moreover, the selected semiconductor components should satisfy the requirement that one of two contacted semiconductors should have higher CB, VB position and *E*_f_ than the other, whereas whether the constructed photocatalyst follows the direct Z-scheme mechanism or not can be validated by the experimental methods performing expensive instruments. Therefore, the combination theoretical calculation and simulation with experimental characterizations may be a cost-effective strategy to comprehensively understanding the mechanism of Z-scheme and successfully construct direct Z-sheme photocatalysts. (2) Optimizing and controlling the contact interface between the selected semiconductor components of a direct Z-scheme photocatalyst is necessary for high efficiency of charge carrier separation and transfer which is beneficial to enhance the photocatalytic performance of prepared direct Z-scheme photocatalysts. (3) For specific photocatalytic applications, introducing suitable, abundant, and inexpensive co-catalysts into the Bi-based Z-scheme semiconductor will further improve the efficiency of photocatalytic applications. In addition, modulating the morphology of the Bi-based Z-scheme semiconductor to obtain high specific surface area is another way to enhance the efficiency of photocatalytic applications owing to this providing more active sites for the reactive matters. (4) In practical applications, it is also necessary to consider the recovery of Bi-based Z-scheme photocatalysts. In most applications, the photocatalyst is dispersed in the solution in the form of powders, which makes recovery work after application very complicated. Thus, constructing Bi-based Z-scheme photocatalysts with magnetic or self-floating properties is a new direction.

Owing to the potential of the Bi-based Z-scheme photocatalyst, it would be promising to realize its industrialization after resolving these challenges.

## Figures and Tables

**Table 1:** Comparison of extensively used synthesis methods of direct Z-scheme photocatalysts.

Synthesis Method	Advantages	Shortcomings	Interfacial Properties of the Prepared Z-Scheme Hetero- Junction	Geometrical Configurations of the Prepared Z-Scheme Heterojunction
Hydrothermal and Solvothermal Method	Controllable Size, High Crystallinity, Low Cost, Simple Operation, One-Pot Synthesis without Need of Post Annealing	High Requirements in Temperature, Pressure and Corrosion Resistance for Equipment, Required High Temperature	Strong Interaction and Intimate Interface	Surface-Decorated Structure
Solid-State Synthesis	High Synthetic Efficiency, Simple and Solvent-Free Synthetic Process	High Energy Consumption, High Cost, Required High Temperature	Strong Interaction and Tight-Contact Interface	Surface-Decorated Structure
Deposition- Precipitation Method	Narrow Size Distributions of Products, Good Thermal Stability of Products	Poor Reproducibility, Uncontrollable Deposition Location and Nucleation Site	Strong Interaction and Intimate Interface	Surface-Decorated Structure
Cation Exchange Method	Relatively Rapid Reaction Rate, Well-Preserved Initial Morphology, Size and Compositional Interfaces, High-Quality Nanocrystal, Simple and Flexible Method	Required Post Calcination Treatment	Strong Interaction, High-Quality and Atomic- Precision Contact Interface	Janus, Surface-Decorated or more Complex Custom Structure Including Multicomponent Z-Scheme Heterojunction Structure
Electro- Spinning Method	Facile and Simple Method, Simple Setup, Large Surface Area of Products	Low Synthetic Efficiency, High Cost, Required Post-Heating Treatment	Strong Interaction and Intimate Interface	Surface-Decorated Structure
Self- Assembly Method	Mild Operation Conditions, Controllable Morphology and Size, Highly Ordered and Dispersive Products	Low Yield, Poor Stability of Products	Moderate Interaction	Core-Shell, Surface-Decorated Structure
Mechanical Agitation Method	Simple Setup, Straightforward Method, Avoiding the Use of Complex and Tedious Chemical and Thermal Treatments	Wide Size Distributions of Products, Poor Reproducibility, Uncontrollable Size	No Intimate Interface, Having Easily Detachable Components of Heterojunction, Low Crystallinity	Surface-Decorated Structure
Ultrasonic Chemical Method	Narrow Size Distributions of Products, Rapid Reaction Rate, Controllable Morphology and Size	High Cost, Hard to Scaling Up	Strong Interaction and Intimate Interface	Surface-Decorated or Core-Shell Structure

**Table 2:** Research progress on Bi-based Z-Scheme photocatalysts applied in photocatalytic degradation.

Photo- Catalyst	Synthesis Method	Light Source	Catalyst Dose	Pollutants	Photocatalytic Efficiency	Ref.
Bi_2_WO_6_/CuBi_2_O_4_	Hydro- Thermal	300 W Xe Lamp (λ ≥ 400 nm)	0.5 mg/mL	Tetracycline (15 mg/L, 100 mL)	0.0393 min^−1^ (CuBi_2_O_4_ 0.0054 min^−1^)	[70]
BiOI /g–C_3_N_4_	In situ Reduction and Oxidiza- tion	60 W LED (Light Emitting Diode) Lamp (λ > 400 nm)	3.33 mg/mL	Phenol (100 mg/L, 15 mL)	60% (BiOI 20%)	[76]
CdS/BiOI	Hydro- Thermal	300 W Xe Lamp (λ > 420 nm)	0.2 mg/mL	RhB (20 mg/L, 100 mL)	0.03945 min^−1^ (BiOI 0.00398 min^−1^)	[77]
BiOBr/ Bi_2_MoO_6_	Co-Precipitation	300 W Xe Lamp (λ ≥ 420 nm)	0.2 mg/mL	Cipro- Floxacin (10 mg/L 50 mL). RhB (10^−5^ mol/L, 50 mL)	84.63% (Bi_2_MoO_6_ 15.21%); 0.37613 min^−1^ (Bi_2_MoO_6_ 0.00689 min^−1^)	[78]
Bi_2_O_3_/g–C_3_N_4_	Solid- State Synthesis	500 W Xe Lamp (λ > 400 nm)	1.0 mg/mL	MB (1.1 × 10^−5^ mol/L, 300 mL); RhB (1.0 × 10^−5^ mol/L, 300 mL)	0.0253 min^−1^ (g–C_3_N_4_ 0.0074 min^−1^); 0.0101 min^−1^ (g–C_3_N_4_ 0.002 min^−1^)	[79]
Bi_2_Fe_4_O_9_/Bi_2_WO_6_	Hydro- Thermal	300 W Xe lamp (λ ≥ 420 nm)	0.3 mg/mL	RhB (10 mg/L, 100 mL)	0.0380 min^−1^ (Bi_2_Fe_4_O_9_ 0.0015 min^−1^)	[74]
AgI/Bi_5_O_7_I	Ion Exchange	350 W Xe lamp (cut off UV and IR light)	1.0 mg/mL	RhB (10 mg/L, 100 mL)	0.046 min^−1^ (Bi_5_O_7_I 0.012 min^−1^)	[80]
AgI/Bi_2_WO_6_	Precipitation	300 W Xe lamp (λ ≥ 420 nm)	0.3 mg/mL	Tetracycline (20 mg/L, 100 mL)	0.075 min^−1^ (Bi_2_WO_6_ 0.014 min^−1^)	[81]
AgBr/CuBi_2_O_4_	Precipitation	300 W Xe lamp (λ ≥ 420 nm)	0.5 mg/mL	Tetracycline (10 mg/L, 100 mL)	0.03551 min^−1^ (CuBi_2_O_4_ 0.00238 min^−1^)	[82]
TCPP/rGO/Bi_2_WO_6_	Ultrasonic Chemical	300 W Xe lamp (λ > 420 nm)	0.3 mg/mL	Tetracycline (15 mg/L, 100 mL)	83.60% (Bi_2_WO_6_ 48.61%)	[83]
Ag_3_PO_4_/CuBi_2_O_4_	Precipitation	300 W Xe lamp (λ > 420 nm)	0.5 mg/mL	Tetracycline (10 mg/L, 100 mL)	0.0201 min^−1^ (CuBi_2_O_4_ 0.0072 min^−1^)	[84]
Porous g–C_3_N_4_/BiOI	Hydro- Thermal	50 W 410 nm LED light arrays	1 mg/mL	MB (20 mg/L, 30 mL)	0.0160 min^−1^ (BiOI 0.0041 min^−1^)	[85]
CdS/Bi_3_O_4_Cl	Hydro- Thermal	250 W Xe lamp (λ > 420 nm)	0.5 mg/mL	Tetracycline (10 mg/L, 100 mL). Cipro-Floxacin (10 mg/L, 100 mL)	0.0643 min^−1^ (Bi_3_O_4_Cl 0.0148 min^−1^). 0.0151 min^−1^ (Bi_3_O_4_Cl 0.00142 min^−1^)	[75]
Cu_2_O/Bi_5_O_7_I	Glucose Reduction Reaction	500 W Xe lamp	1 mg/mL	RhB (10 mg/L, 100 mL)	0.0233 min^−1^ (Bi_5_O_7_I 0.00736 min^−1^)	[86]
CuInS_2_/Bi_2_WO_6_	Hydro- Thermal	300 W Xe lamp (λ ≥ 420 nm)	0.3 mg/mL	Tetracycline Hydrochloride (10 mg/L, 100 mL)	0.0176 min^−1^ (Bi_2_WO_6_ 0.01473 min^−1^)	[87]
MoO_3_/Bi_2_O_4_	Hydro- Thermal	100 W LED lamp (λ = 420 nm)	0.5 mg/mL	RhB (10 mg/L, 100 mL)	99.6% (Bi_2_O_4_ 73%)	[88]
BiOI/Bi_2_O_4_	Ultrasonic Chemical	100 W LED lamp	0.5 mg/mL	RhB (10 mg/L, 100 mL)	0.090 min^−1^ (BiOI 0.003 min^−1^)	[89]
Bi_2_MoO_6_/TiO_2_	Hydro- Thermal	800 W Xe lamp	0.6 mg/mL	4-Nitrophenol (50 mg/L, 100 mL)	95.3% (Bi_2_MoO_6_ 32.7%)	[90]
Bi_2_WO_6_ /Porous g–C_3_N_4_	Ultrasonic Chemical	500 W Wolfram lamp (λ ≥ 420 nm)	0.5 mg/mL	RhB (10 mg/L, 100 mL)	0.043 min^−1^ (Bi_2_WO_6_ 0.013 min^−1^)	[91]
Bi_2_WO_6_/BiOI	Hydrothermal	500 W Xe lamp (λ > 420 nm)	1 mg/mL	RhB (10 mg/L, 40 mL)	0.03 min^−1^ (BiOI 0.002 min^−1^)	[92]
Ag_3_PO_4_/Bi_2_WO_6_	Precipitation	50 W LED lamp (λ = 410 nm)	1 mg/mL	MB (20 mg/L, 30 mL)	0.61 min^−1^ (Bi_2_WO_6_ 0.10 min^−1^)	[93]
g–C_3_N_4_/BiVO_4_	Hydrothermal	250 W Xe lamp (λ > 420 nm)	0.2 mg/mL	MO (20 mg/L, 50 mL)	0.09672 min^−1^ (BiVO_4_ 0.01101 min^−1^)	[94]

**Table 3:** Research progress of Bi-based Z-Scheme photocatalyst applied in solar-water splitting.

Photo- Catalyst	Co- Catalyst	Synthesis Method	Experimental Conditions	Products and Yields	AQY	Ref.
BiPO_4_/SnS_2_	No	Hydrothermal	Visible light irradiation (λ > 380 nm). Pure Water	H_2_: 303 μmol h^−1^·g^−1^	–	[98]
Bi/Bi_5_O_7_I/Sn_3_O_4_	Bi	Hydrothermal	300 W Xe Lamp (λ > 400 nm). 20% CH_3_OH Solution	H_2_: 325.9 μmol h^−1^·g^−1^	–	[99]
Cu_3_P/Bi_2_WO_6_	No	Mechanical Agitation	Xe lamp (AM (air mass) 1.5); 0.5 M Na_2_HPO_4_/NaH_2_PO_4_ Solution	H_2_: 4.65 μmol h^−1^·g^−1^ O_2_: 2.3 μmol h^−1^·g^−1^	–	[68]
BiVO_4_ /Black phosphorus	5 wt% Co_3_O_4_	Self-Assembly	320 W Xenon Lamp (λ > 420 nm). Pure Water	H_2_: 160 μmol h^−1^·g^−1^ O_2_: 102 μmol h^−1^·g^−1^	0.89% at 420 nm	[100]
Bi_2_O_2.33_/Bi_2_S_3_	1 wt% Pt	Wet Chemistry	500 W Xenon Lamp; 0.1 M Na_2_S/ Na_2_SO_3_ Solution	H_2_: 62.61 μmol h^−1^	–	[101]
g–C_3_N_4_ /BiFeO_3_	No	Solid-State Synthesis	Three 125 W Medium Pressure Hg Lamps (UV). Pure Water	H_2_: 160.75 μmol h^−1^·g^−1^ O_2_: 80.12 μmol h^−1^·g^−1^	–	[102]
CdS/BiVO_4_	2 wt% Pt	Solvothermal	300 W Xe Lamp (λ ≥ 420 nm); 20 vol.% Lactic Acid Solution	H_2_: 1153 μmol h^−1^	–	[103]
Bi_2_S_3_/MoS_2_/TiO_2_	No	Microwave- Assisted Hydrothermal	250 W Xe Lamp (λ ≥ 420 nm); 0.35 M Na_2_S and 0.25 M Na_2_SO_3_ Solution	H_2_: 2195 μmol h^−1^·g^−1^	–	[104]
Cs_2_O/Bi_2_O_3_/ZnO	No	Solution Combustion Method	Xe Lamp (AM 1.5 G); Pure Water	H_2_: 149.5 μmol h^−1^·g^−1^ O_2_: 73.2 μmol h^−1^·g^−1^	1.68% at 365 nm 0.92% at 420 nm	[105]
ZnIn_2_S_4_/RGO/BiVO_4_	1 wt% Pt	Hydrothermal	350 W Xe Lamp (λ > 420 nm); 5 mol·L^−1^ HCHO	H_2_: 1687 μmol h^−1^·g^−1^	22.91%	[106]
RGO–Cu_2_O/Bi_2_WO_6_	No	Solvothermal	Xe Lamp (λ > 420 nm); Pure Water	H_2_:1.80 μmol h^−1^·g^−1^	–	[107]
Cu_2_O/RGO/BiVO_4_	–	Solvothermal	300 W Xenon Arc Lamp (λ > 420 nm); TC Solution	H_2_: 5.90 μmol h^−1^·g^−1^	–	[108]

**Table 4:** The research progress of Bi-based Z-scheme photocatalyst in CO_2_ reduction.

Photo- Catalyst	Co- Catalyst	Synthesis Method	Conditions	Products and Yields	Ref.
CdS/BiVO_4_	No	Deposition	300 W Xenon Arc Lamp (λ > 400 nm). 20 mg Photocatalyst in 180 mL Stainless Steel Reactor with Quartz Window; Filled with CO_2_ (0.3 MPa).	CH_4_: 1.75 μmol h^−1^·g^−1^ CO: 0.39 μmol h^−1^·g^−1^	[115]
BiOI/g–C_3_N_4_	No	Deposition	300 W Xenon Arc Lamp (λ > 400 nm); 0.1 g Photocatalyst in 180 mL Stainless Steel Cylindrical Vessel with Quartz Window; Introducing CO_2_ and H_2_O Vapor by Bubbling Approach.	CH_4_: 1.76 μmol h^−1^·g^−1^ CO: 22.21 μmol h^−1^·g^−1^ H_2_: 2.06 μmol h^−1^·g^−1^ O_2_: 10.81 μmol h^−1^·g^−1^	[116]
Bi_2_WO_6_/TiO_2_	No	Electrostatic Self- Assembly	300 W Xenon Arc Lamp (780 nm > λ > 320 nm); 20 mg Photocatalyst in 25 mL Quartz Reactor; CO_2_ was Evacuated by a Mechanical Pump.	CH_4_: 10.8 μmol h^−1^·g^−1^ CO: 25.8 μmol h^−1^·g^−1^	[117]
g–C_3_N_4_/ Bi_2_O_2_[BO_2_(OH)]	No	Solid-State Synthesis	300 W Xe Lamp; 20 mg Photocatalyst; 1.7 g Na_2_CO_3_ Treated with 15 mL H_2_SO_4_ (0.1 mol/L) to in situ Generate CO_2_.	CO: 6.09 μmol h^−1^	[118]
Bi_2_WO_6_/g–C_3_N_4_	No	Hydrothermal	300 W Xenon Arc Lamp (λ > 420 nm); 100 mg Catalyst in 500 mL Reactor; Introducing CO_2_ and H_2_O Vapor by Bubbling Approach.	CO: 5.19 μmol h^−1^·g^−1^	[119]
Bi_4_TaO_8_Cl /W_18_O_49_	No	Solvothermal	180 mW/cm^2^ Solar Light (λ < 780 nm); 0.02 g Photocatalyst and 2 mL H_2_O in Reactor; Filled with CO_2_. The Reactor was Heated to 393 K.	CO: 23.42 μmol h^−1^·g^−1^	[120]
Bi_2_O_2_CO_3_/Bi/ Bi_2_WO_6_	Bi	Solvothermal	300 W Xe Lamp; 0.1 g Photocatalyst and 100 mL H_2_O in Reactor; CO_2_ was Inflated into the Reactor (80 kPa).	CH_4_: 2.54 μmol h^−1^·g^−1^ CO: 0.82 μmol h^−1^·g^−1^	[71]
g–C_3_N_4_/BiOBr	Au	Water Bath	300 W High Pressure Xenon Lamp. 0.1 g Samples were Uniformly Dispersed onto a Glass Sheet put in 350 mL Reactor; 1.3 g NaHCO_3_ reacted with 5 mL H_2_SO_4_ (4M) to in situ Generate CO_2_.	CH_4_: 0.92 μmol h^−1^·g^−1^ CO: 6.67 μmol h^−1^·g^−1^	[121]
Bi_2_WO_6_ /RGO /g–C_3_N_4_	No	Hydrothermal	300 W Xe Arc Lamp with a UV cut-off Filter of 420 nm; 50 mg of the Catalyst was Uniformly Distributed in the Photoreactor (250 mL); A Water Bubbler to generate a Mixture of CO_2_ and Water Vapor.	CO: 15.96 μmol h^−1^·g^−1^ CH_4_: 2.51 μmol h^−1^·g^−1^	[122]
g–C_3_N_4_/Bi_4_O_5_I_2_	No	Complex Precursor Method	300 W High Pressure Xenon Lamp (λ > 400 nm); 0.1 g Samples were Uniformly dispersed onto a Glass Sheet, put in 350 mL Reactor; NaHCO_3_ reacted with 5 mL H_2_SO_4_ (4M) to achieve 1 atm CO_2_.	CO: 45.6 μmol h^−1^·g^−1^	[123]
BiVO_4_/C/Cu_2_O	No	SILAR	300 W Xe Lamp (λ > 420 nm); A 1 cm^2^ Specimen of the Sample was placed at 50 mL Reactor which charged with 5 mL of H_2_O; The Reactor was purged with CO_2_.	CO: 3.01 μmol h^−1^·g^−1^	[124]
